# Demand for CAM Practice at Hospitals in Japan: A Population Survey in Mie Prefecture

**DOI:** 10.1093/ecam/neq049

**Published:** 2010-09-15

**Authors:** Toshihiro Togo, Shigeru Urata, Kenta Sawazaki, Hinata Sakuraba, Torao Ishida, Kazuhito Yokoyama

**Affiliations:** ^1^Department of Acupuncture and Moxibustion, Faculty of Health Sciences, Tokyo Ariake University of Medical and Health Sciences, Tokyo, 135-0065, Japan; ^2^Department of Epidemiology and Environmental Health, Juntendo University Faculty of Medicine, Tokyo, 113-8421, Japan; ^3^Department of Acupuncture Medicine, Faculty of Oriental Medicine, Suzuka University of Medical Science, Suzuka, Mie, 510-0293, Japan; ^4^Department of Acupuncture and Moxibustion Therapy, Faculty of Health Promotional Sciences, Hamamatsu Unviersity, Hamamatsu, Shizuoka, 431-2102, Japan; ^5^Department of Health, Faculty of Health Sciences, Tsukuba University of Technology, Tsukuba, Ibaragi, 305-8521, Japan; ^6^Institute of Traditional Chinese Medicine, Suzuka University of Medical Science, Suzuka, Mie, 510-0293, Japan

## Abstract

Complementary and alternative medicine (CAM) therapies have been provided at hospitals along with conventional medicine in industrialized nations. Previous studies conducted in Japan revealed high proportion of Japanese had experience of using CAM, but failed to discuss how it should be provided. The present study aims to clarify the demand for CAM practice at hospitals in Japan. A questionnaire consisting of 41 questions was mailed to 10 000 adults randomly selected from the electoral roll of Mie prefecture, Japan in January 2007. The questionnaire asked the subjects about demand for CAM practice at hospitals, types of CAM therapy to be provided and associated reasons. Sociodemographic characteristics, perceived health status, experience and purpose of CAM use, and information resource for CAM were also surveyed. Completed answers were collected from 2824 (28.6%) respondents. Two thousand and nineteen (71.5%) of the respondents demanded CAM practice at hospitals with the most likely reason of “patients can receive treatment under the guidance of a physicians". The three most popular CAM therapies were Kampo, acupressure/massage/Shiatsu and acupuncture/moxibustion. The demand was positively associated with gender, ages of 40–59 years, annual household incomes of 5–7 million yen, occupation of specialist and technical workers and sales workers and poor health status. Higher demand was observed among those who used both CAM and conventional medical therapies for curative purposes. In conclusion, Japanese show a high demand for CAM practice, hoping to use CAM for curative purposes with monitoring by physicians at hospitals.

## 1. Introduction

With the increasing practice of complementary and alternative medicine (CAM), a bulk of studies including nationwide surveys have revealed levels of use of CAM therapy and characteristics of its users in industrialized nations [1–9]. Studies on the cost effectiveness have also presented evidence that CAM therapy can potentially contribute to a reduction in medical expenditure [10, 11]. Although dependent on social factors, a number of hospitals in industrialized nations have begun to integrate CAM practice into the conventional medical service. For example, in the USA, >37% of hospitals had introduced one or more CAM therapies in 2008 [12]. In Japan, hospitals including those allied with national universities have begun to establish departments for providing CAM practice in recent years. The practice of CAM, however, is often accompanied by challenges such as lack of licensure or education system, lack of insurance coverage and insufficient scientific evidence. Only a limited number of physicians are ready to coordinate with CAM practitioners [13].

In the west, with growing interest in integrative medicine, many research have been conducted focusing on credentialing CAM provider [14], the present situation of integration of CAM into conventional medical settings [15–18], CAM education at medical schools [19–22] and communication between physicians and CAM practitioners at hospitals [23–25]. On the contrary in Japan, most of the previous studies dealt with the popularity of CAM therapies and the backgrounds of users [7, 8], but few have discussed how and where people want them to be provided. As a wide range of legislation reformation will be required, if people want to receive CAM at hospitals along with conventional medicine, it is important to obtain information on the demand. In the present study, a population survey is conducted to assess the demand for CAM practice, reasons for the demand, types of CAM therapy to be provided and expected purposes for CAM at hospitals in Japan.

## 2. Methods

A total of 10 000 people, aged 20 years or above, were randomly selected from the electoral roll of all 29 cities and towns in Mie Prefecture. Sampling was conducted according to the geographic proportion of the population and in accordance with the Public Office Election Law of Japan. A questionnaire was mailed to the recipients on January 9, 2007, asking them to answer questions anonymously and to return it by January 31. It did not include any question on the clinical data that would lead to the identification of respondents. In the cover letter attached to the questionnaire, recipients were informed that the personal data including name and address were used only for sending the questionnaire, that the data indicated in the response sheets would not be used for purpose other than research, and that none of the data would not be transmitted to other parties.

Fifteen types of therapy (Table 1) were given as complementary and alternative therapies and the subjects were asked “Do you want to receive CAM therapy at hospitals?" (i.e., demand for CAM practice at hospitals). Respondents who gave “Yes" to this question were requested to answer which CAM therapy they wanted to be provided at hospitals along with conventional medicine and their reasons. The questionnaire also inquired on their experience of CAM therapy over a lifetime as well as conventional medical therapy within the last 12 months at their hospitals. Those who experienced CAM therapy (CAM users) were requested to report the purposes and information resources for the therapy. If the respondents reported they had no experience of CAM therapy or had no desire to receive it (non-CAM users), they were asked to give the reasons. Sociodemographic characteristics (age, gender, area of residence, occupation and annual household income) and perceived health status were asked in the questionnaire.

The chi-square (*χ* ^2^) test was conducted to determine whether there was an association of the demand for CAM practice at hospitals with demographic characteristics, perceived health status and purpose for CAM use. Adjusted standardized residuals in each cell of contingency tables were examined to assess the significance of association [26]. Analysis was performed using PASW Statistics version 18.0 for Windows.

## 3. Results

### 3.1. Response Rate

A total of 2824 questionnaires were collected before January 31, with 112 undelivered. Thus, the response rate was 28.6% (2824/9888). CAM was received by 64% (1807) of the respondents over a lifetime, whereas conventional medical therapies were received by 77% (2178) within the last 12 months.

### 3.2. Demand for CAM at Hospitals, Its Reason and the Types of CAM Demanded

The demand for CAM practice at hospitals was reported by 71.5% (2019) of the respondents. The types of CAM therapy that the respondents wanted to be administered are listed in Table 1. The four most in-demand CAM therapies were “Kampo", “acupressure/massage/Shiatsu", “acupuncture/moxibustion" and “seitai/chiropractic". More than half of the respondents gave the reason for their choice as “patients can receive treatment under the guidance of a physician" (Table 2).

### 3.3. Sociodemographic Indicators

Table 3 shows relationships of the demand for CAM practice at hospitals to sociodemographic characteristics and perceived health status. The demand was higher in females than in males. It varied among age groups, that is, it was higher in the 40- to 59-year-old age group, whereas it was lower in the younger (<30 years) and the older (>60 years) age groups. Those whose household income was 5–7 million yen showed a significantly higher demand, whereas those with <3 million yen had a lower demand. Higher demand was observed in “specialist and technical worker" and “sales worker" groups, whereas demand was low in “unemployed", “production process and related workers" and “agriculture, forestry and fishery workers" groups. In all the categorized groups with perceived health status, high demand for CAM practice at hospitals was observed. However, respondents who had both mental and physical problems reported significantly higher demand, whereas those who felt neither of them did have a significantly lower demand.

### 3.4. Purpose of CAM Use at Hospitals

The relationships between the purposes of CAM use and the demand for CAM practice at hospitals in 1807 CAM users are shown in Table 4. Those who received both CAM and conventional medical therapies for curative purposes had a higher demand for CAM practice at hospitals than those who used CAM for preventive or refreshment purposes.

### 3.5. Reasons for Disuse of CAM and Information Resource of CAM

The two most common reasons for disuse of 678 non-CAM users were “unclearness of the effect of CAM" and “lack of CAM therapy knowledge" (Table 5). As for information resource for CAM, 77% (1404) of CAM users depended on their family or an acquaintance for their information for CAM. Other resources are as follows: “books/magazines" (550, 29%), “TV/radio programs" (468, 25%) and “recommendation by a physician" (335, 18%).

## 4. Discussion

It was found that over 70% of the respondents reported that they want to receive CAM therapy at hospitals. The three most in-demand CAM therapies at hospitals with conventional medicine were Kampo, acupressure/massage/Shiatsu and acupuncture/moxibustion, followed by seitai/chiropractics. Among them, Kampo is already being practiced by physicians as a part of conventional medicine in Japan. Hence, according to the classification by National Centre for Complementary and Alternative Medicine (NCCAM), it is possible that the Japanese want manipulative and body-based methods mostly to be administrated at hospitals. As shown in Table 6, under the current Japanese healthcare system, acupressure/massage/Shiatsu and acupuncture/moxibustion, both of which have a long history of practice in Japan as a part of traditional medicine, are practiced with a national license, but chiropractics and seitai lack such regulation or an education system. If those therapies need to be provided along with conventional medicine in Japan, legislative reformation is required.

Previous studies revealed that 55–62% of the patients who received CAM therapy did not report it to their physicians, suggesting that physicians should pay more attention to their patients' CAM use [4, 5, 7, 8]. In the present study, more than half of the respondents indicated the presence of a physician's guidance as the reason for demanding CAM practice at hospitals (Table 2), indicating that they placed reliance on physicians in using CAM. Thus, CAM may play a complementary role in medical care, under recognition by physicians.

Nearly half of non-CAM users answered “lack of knowledge on CAM" as the reason for not using CAM in the present study. Given the fact that over three-quarters of the CAM users obtain the information from their family or an acquaintance, CAM users also might lack sufficient information about the indications or effects of each CAM therapy. This suggests an anxiety about lack of knowledge on CAM may be associated with the expectation for an environment in which they can receive advice from physicians. Among the CAM users, those who received both CAM and conventional medical therapies for curative purposes are most likely to demand CAM practice at hospitals, whereas those who received only CAM for refreshment showed lower demand, suggesting that more people hope to see that CAM therapies are provided as a curative measure rather than as preventive one or refreshment.

The combined practice of CAM therapy and conventional medicine is defined as integrative medicine by NCCAM as “integrative medicine combines conventional and CAM treatments for which there is evidence of safety and effectiveness [27]". Besides scientific evidence, the practice of integrative medicine would require a wide range of legislation reforms such as a licensure system for CAM, education curricula in medical schools and insurance systems in accordance with the medical systems of each nation. The present survey demonstrated that many Japanese wanted CAM therapies such as Kampo and manipulative and body-based methods to be integrated into medical service at hospitals. However, the current healthcare system in Japan offers only a minimum of 8-h training for Kampo or acupuncture in a 6-year education program in medical schools, which is far from sufficient; less education is provided to physicians for other types of CAM. With little knowledge on indication or possible adverse effects, physicians are not ready to use CAM or to coordinate with CAM practitioners. Another fundamental barrier to integrating CAM into conventional medical service is the current public health insurance system, which prohibits a patient to have reimbursed both for conventional medical treatment and CAM therapy, if the patient receives those therapies at a hospital under the same diagnosis. Thus, reforms in public health insurance system, as well as educational program are important when CAM therapies are integrated.

Higher demand for CAM practice at hospitals was observed in females, the groups of those aged 40–59 years, in annual household incomes of 5–7 million yen, and in specialist and technical workers and sales workers. Those who reported both mental and physical problems showed higher demand. These results, together with the observation of lower demand among those aged >60 years, those with their annual income over 7 million yen and those engaged in administrative and management works, suggest that people from middle classes and those engaged in intellectual occupation but not in administrative position, were the most likely users of CAM provided at hospitals.

**Figure 1:**
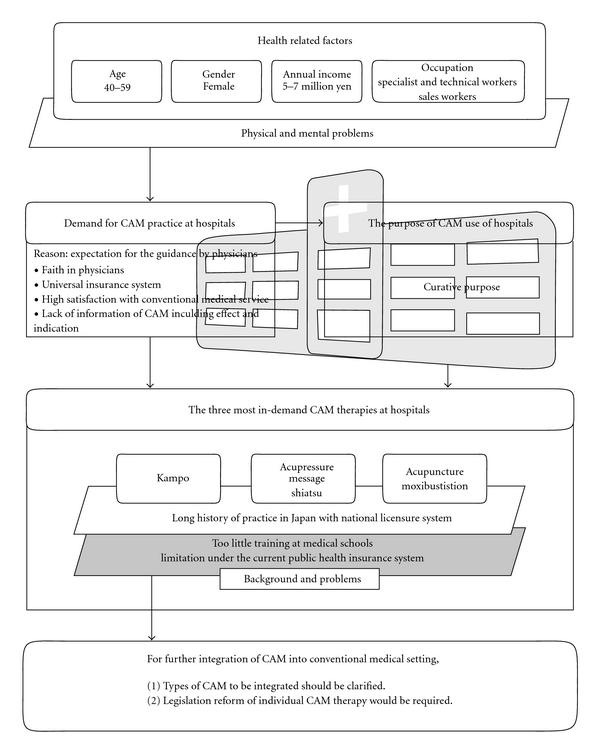
Background on the demand for CAM therapy at hospitals in Japan and implications for future study.

The present study demonstrated high demand for CAM practice at hospitals in Japan. However, this does not mean that all CAM therapies are to be included in the medical service at hospitals, however. For better integration of CAM with conventional medicine at hospitals, further surveys should be conducted to make clear which CAM therapies are to be administered and which are to be provided independent of conventional medical service at hospitals. The background on the demand for CAM practice at hospitals and the implications for future study are summarized in Figure 1. 

Finally, the present survey has the following limitations. First, the study was conducted by carrying out a postal survey and the response rate was relatively low compared with other surveys conducted in western nations. There was no information on the non-respondents. Also, recipients who were hospitalized might not have answered the questionnaire. Second, the respondents were not asked about the seriousness of their illness. Third, geographical characteristics of the sampling population should be considered. Mie prefecture is a relatively rural state located south of Kyoto and Nagoya, with a population of 1 867 696 (the population of adults aged 20 years or above is 1 503 662) [28], and the low prevalence of CAM therapies such as reflexology and yoga, which are relatively new to the Japanese population, may have influenced the respondents' CAM knowledge.

Despite those limitations, however, sampling population of our survey is not considered to be more likely to include people in favor of CAM. In our survey, 23.7% (669/2824) of the respondents reported lifetime experience of acupuncture treatment, which is quite similar with the data shown in Ishizaki et al.'s nationwide surveys conducted annually from 2003 through 2006 (26.7, 19.4, 24.4 and 25.4, resp.) [9]. Our study showed the lifetime utilization of Kampo is 37.5% (1058/2824), which is also close to the figure shown in the Okabe's survey (*∼*40%) conducted at four major general hospitals in Tokyo [29].

## 5. Conclusion

In Mie prefecture, Japan, there is a high demand for CAM practice, especially Kampo and manipulative methods, along with conventional medicine as a curative measure with monitoring of physicians at hospitals. These findings should be confirmed through nationwide survey and can be utilized for better integration of CAM into conventional medical setting in Japan.

## Funding

This work was financially supported by the Ministry of Education, Culture, Sports, Science and Technology of Japan.

## Figures and Tables

**Table 1:** Types of CAM therapy 2019 respondents wanted to be provided at hospitals (Number with percent in parenthesis).

Type of CAM	Number (%)
Kampo	1391 (69.0)
Acupressure, massage and Shiatsu	1157 (57.4)
Acupuncture and moxibustion	815 (40.4)
Seitai^(a)^ or chiropractic	778 (38.6)
Psychotherapy	544 (27.0)
Balneotherapy	530 (26.3)
Health foods (supplement)	477 (23.6)
Reflexology	370 (18.3)
Aromatherapy	237 (11.8)
Yoga (Ayurveda)	235 (11.7)
Qigong	222 (11.0)
Music therapy	206 (10.2)
Hypnotherapy	159 (7.9)
Meditation	98 (4.9)
Thalassotherapy	90 (4.5)
Others	10 (0.5)

^(a)^Seitai is a kind of manipulative technique originated in Japan, which is practiced mainly for joint adjustment.

**Table 2:** Reasons for the demand for CAM practice at hospitals in 2019 respondents (Number with percent in parenthesis).

Reason	Number (%)
Patients can receive treatment under guidance of a physician	1100 (54.5)
Health insurance may be used	984 (48.8)
Patients can receive both CM and CAM in a single hospital	950 (47.1)
Higher effect can be expected by combining CM and CAM	858 (42.5)
Multiple approaches to illness can be expected	854 (42.3)
Better sanitary condition can be expected	142 (7.0)
Other	30 (1.5)

CM, conventional medicine.

**Table 3:** Relations between sociodemographic characterisitics, perceived health status and demand for CAM practice at hospitals among all 2824 respondents.

	Do you want to receive CAM at hospitals?		Adjusted standardized residuals	*P*-value (*χ* ^2^ test)
	Yes (%)	No (%)		
Gender^(a)^				
Male	850 (74.1)	297 (25.9)	−2.9	<.05
Female	1118 (78.9)	299 (21.1)	2.9	
Age (years)^(b)^				
20–29	185 (72.0)	72 (28.0)	−2	<.001
30–39	352 (80.2)	87 (19.8)	1.7	
40–49	463 (82.2)	100 (17.8)	3.3	
50–59	545 (80.6)	131 (19.4)	2.6	
60–69	343 (69.0)	154 (31.0)	−4.7	
>70	115 (67.6)	55 (32.4)	−3	
Annual household income (yen)^(c)^				
<3 million	398 (70.3)	168 (29.7)	−4.2	<.001
3–5 million	525 (77.1)	156 (22.9)	0.2	
5–7 million	410 (81.8)	91 (18.2)	2.9	
7–10 million	343 (76.9)	103 (23.1)	0	
>10 million	226 (80.7)	54 (19.3)	1.6	
Occupation^(d)^				
Specialist and technical workers	338 (81.3)	78 (18.8)	2.2	<.001
Administrative and managerial workers	139 (79.4)	36 (20.6)	0.8	
Clerical workers	220 (81.2)	51 (18.8)	1.7	
Production process and related workers	164 (71.6)	65 (28.4)	−2	
Service workers	165 (77.1)	49 (22.9)	0	
Security workers	14 (82.4)	3 (17.6)	0.5	
Transport and communication workers	36 (78.3)	10 (21.7)	0.2	
Agriculture, forestry and fishery workers	45 (59.2)	31 (40.8)	−3.7	
Sales workers	100 (87.0)	15 (13.0)	2.6	
Student	33 (73.3)	12 (26.7)	−0.6	
Housewife/househusband	426 (79.5)	110 (20.5)	1.5	
Not employed	231 (67.2)	113 (32.8)	−4.7	
Other	90 (78.9)	24 (21.1)	0.5	
Perceived health status				
Both mentally and physically fit	747 (74.0)	262 (26.0)	−2.9	<.05
Physically fit but mentally some problems	180 (76.9)	54 (23.1)	0	
Mentally fit but physically have some problems	643 (77.3)	189 (22.7)	0.2	
Both mentally and physically have problems	438 (82.2)	95 (17.8)	3.2	

*P*-values indicate differences in proportion between gender, age groups, annual income groups, occupational groups and “perceived health status" groups, respectively.

^(a)^260 (9.2%); ^(b)^222 (7.9%); ^(c)^350 (12.4%); and ^(d)^226 (8.0%) of the respondents did not answer the questions, respectively.

**Table 4:** Relations between the purpose for CAM use and demand for CAM practice at hospitals in 1807 CAM users.

Purpose of CAM use	Do you want to receive CAM at hospitals?		Adjusted standardized residuals	*P-*value (*χ* ^2^ test)
	Yes (%)	No (%)		
Curative purpose (also use conventional medicine)	545 (90.5)	57 (9.5)	4.8	<.001
Curative purpose (only use CAM therapy)	280 (85.6)	47 (14.4)	0.5	
Preventive purpose	217 (81.6)	49 (18.4)	−1.6	
Refreshment	448 (79.7)	114 (20.3)	−4.1	

*P*-values indicate differences in the proportions between the four groups. Of 1807 CAM users, 50 respondents did not answer the question.

**Table 5:** Reasons for disuse of CAM therapy in 678 non-CAM users (Number with percent in parenthesis).

Reason	Number (%)
Unclearness of effect of CAM	370 (54.5)
Lack of knowledge about CAM	289 (42.6)
High cost of CAM	241 (35.5)
Conventional medicine alone is satisfactory	178 (26.2)
Have no time to visit CAM practitioner	136 (20.0)
Poor safety and sanitary condition of CAM	70 (10.3)
Adverse effect of CAM	47 (6.9)
Afraid of bad chemistry with CAM practitioner	45 (6.6)
Have dislike of body contact	41 (6.0)
Other	55 (8.1)

**Table 6:** CAM therapies under the current Japanese healthcare system.

Type	National qualification	Health insurance coverage	Education
Kampo	No	Yes^(a)^	A minimum of 8-h training at medical school
Acupuncture and moxibustion	Yes	Limited^(b)^	Three years education at college or
			Four years education at university
Acupressure, massage and Shiatsu	Yes	No	Three years education at college
Seitai	No	No	None
Chiropractic	No	No	None
Aromatherapy	No^(c)^	No	None
Reflexology	No	No	None
Yoga (Ayurveda)	No^(d)^	No	None
Qigong	No	No	None
Health foods	No	No	None
Hypnotherapy	No^(d)^	No	None
Music therapy	No^(d)^	No	None
Meditation	No	No	None
Psychotherapy	No^(e)^	No	None
Balneotherapy	No	No	None
Thalassotherapy	No	No	None

Kampo and balneotherapy have no national licensure system, but medical doctors who have completed 5 years of special training and have passed an examination conducted by the academic society of the respective fields are certificated as medical experts in the field.

^(a)^One hundred and forty-eight Kampo formulae are included under physician's prescription.

^(b)^Limited to six diseases. Consent by physicians is required.

^(c)^Academic society provides society certification for medical doctors or other licensed co-medical practitioners who meet the requirement and passed written examination. There are also qualifications offered by non-academic associations for non-licensed and licensed health professionals.

^(d)^Applicants who meet the requirements of academic society in the field and pass the examinations are provided qualification by the society.

^(e)^Applicants who meet the requirements of foundation in the field and pass the examinations are provided qualification by the foundation.
